# Delay in Presentation After an Acute Stroke in a Multiethnic Population in South London: The South London Stroke Register

**DOI:** 10.1161/JAHA.112.001685

**Published:** 2012-06-22

**Authors:** Juliet Addo, Salma Ayis, Josette Leon, Anthony G. Rudd, Christopher McKevitt, Charles D.A. Wolfe

**Affiliations:** King's College London, Division of Health and Social Care Research, London, United Kingdom (J.A., S.A., J.L., A.G.R., C.M., C.D.A.W.); Stroke Unit, Guy's and St. Thomas' NHS Foundation Trust, St. Thomas' Hospital, London, United Kingdom (A.G.R.); National Institute for Health Research Comprehensive Biomedical Research Centre, Guy's and St. Thomas' NHS Foundation Trust, London, United Kingdom (J.A., C.D.A.W.)

**Keywords:** stroke, prehospital delay, ethnicity, associated factors

## Abstract

**Background:**

Delayed presentation to hospital after an acute stroke is a major explanation given for low thrombolysis rates. This study aimed to investigate the factors associated with delays in presentation after an acute stroke and changes after a mass media campaign.

**Methods and Results:**

Data were from a population-based study involving 1392 patients with first-ever strokes between 2002 and 2010 in a multiethnic South London population. Associations were determined between prehospital delay (≥3 hours) and variables of interest, including ethnicity, by using multivariate logistic regression analyses. Differences in prehospital delay and thrombolysis rates were determined for the period immediately before and after the FAST mass media campaign (2007/2008 versus 2009/2010).

The median (Q_1_ to Q_3_) time to presentation was 4.73 (1.55 to 12.70) hours, and 550 (39.5%) presented within 3 hours of symptom onset. In multivariate analysis, patients of black ethnicity had increased odds of delay (odds ratio: 1.63; 95% confidence interval, 1.11 to 2.38), whereas those with more severe strokes characterized by a higher National Institutes of Health Stroke Scale score (odds ratio: 0.35; 95% confidence interval, 0.20 to 0.61) had reduced odds of delay. There was no difference in the proportion of patients who arrived within 3 hours (*P*=0.30) in the period immediately before and after the FAST campaign (40.7% in 2007/2008 versus 44.9% in 2009/2010). Among patients with ischemic stroke, 119 (11.0%) received thrombolysis between 2002 and 2010, with no difference observed between the pre- and postcampaign periods (16.9% versus 16.4%).

**Conclusion:**

Significant delays in seeking care after stroke still occur in this population despite efforts to increase public awareness. Future educational programs must identify and specifically address factors that influence behavior and should target those at higher risk of delay. **(*J Am Heart Assoc*. 2012;1:e001685 doi: 10.1161/JAHA.112.001685.)**

## Introduction

Thrombolysis with recombinant tissue plasminogen activator (rt-PA) administered up to 4.5 hours after the onset of symptoms has been demonstrated to significantly improve clinical outcomes in patients with acute ischemic stroke.^1,2^ Previously reported thrombolysis rates range from 1.6% to 18%, and these rates have been shown to vary by several factors, including age, sex, stroke severity, and ethnicity.^3–7^ Delayed presentation is a major explanation given for the low thrombolysis rates.^3,7^ The median time from stroke onset to arrival in an emergency department (ED) in a review of at least 48 reports of prehospital delay time, including data from 17 countries, was between 3 and 6 hours.^8^ Patients of black ethnicity have been reported to take longer than white patients to arrive at the ED in some studies^9–11^ but not in others.^12,13^ These previous studies have mainly been conducted in the United States, and ethnic differences in other Western populations remain unexamined. It has been suggested that increasing public awareness of stroke symptoms is likely to decrease prehospital delay and subsequently lead to an improvement in the rates of thrombolysis.^7^ The Department of Health of England launched a campaign (The Stroke: Act FAST (Face, Arms, Speech and Time) awareness campaign) to promote public awareness of stroke in February 2009.^14^ The campaign aimed to educate healthcare professionals and the public on the signs of stroke and the benefits of prompt treatment. It was implemented through a series of advertisements screened on national television over a period of about 1 year, depicting stroke spreading like fire in the brain.

The aims of this study were to examine the time from onset of stroke symptoms to arrival at the ED and factors associated with prehospital delay, including ethnicity, in a multiethnic population in South London between 2002 and 2010. In addition, the study sought to investigate temporal trends in prehospital delay after onset of stroke symptoms and the use of thrombolysis among patients with ischemic stroke in the period before and after the FAST campaign.

## Methods

### Identification of Cases

Patients registered in the South London Stroke Register (SLSR) between January 1, 2002, and December 31, 2010, were included in this study. The SLSR is an ongoing population-based stroke register collecting data on first-ever strokes in patients of all age groups in a defined area of South London. At the 2001 census, the population of the SLSR area was 271 817, with 63% white, 9% black Caribbean, 15% black African, and 13% other ethnic groups. The detailed methods of notification of patients and data collection have been described previously.^15^ In brief, all persons with first-ever strokes notified from multiple sources were enrolled onto the register. A structured questionnaire was completed for every stroke patient by interviewing the patient or family and reviewing their medical notes. Patients with subarachnoid hemorrhage were excluded from this analysis.

### Baseline Assessment

Data obtained on demographic details include age, sex, self-defined ethnic origin (based on the 2001 UK census question and categorized into “white,” “black” [black Caribbean, black African, and black other], and other ethnic groups), and socioeconomic status (based on the Registrar General's occupational codes and grouped into manual, nonmanual, and economically inactive [if student, unemployed, or unable to work because of disability or being a carer]). Patients were examined as soon as possible after notification to the SLSR by a study clinician certified in the use of the National Institutes of Health Stroke Scale (NIHSS),^16^ who recorded the severity of the stroke and neuroradiological features. Stroke severity at the time of maximum impairment was assessed by the Glasgow Coma Score (classified as 0 to 8 and 9 to 15), the NIHSS score (classified in quartiles from the least to the most severe), and the presence or absence of dysphagia. Classification of the pathological subtype (cerebral infarction and primary intracerebral hemorrhage) was based on results from brain imaging (computed tomography scan or magnetic resonance imaging). Cases without pathological confirmation of stroke subtype were considered unclassified.

### Prehospital Delay

Prehospital delay to presentation was defined as the time from the onset of stroke signs or symptoms to arrival at the ED of hospitals serving the study area (2 teaching hospitals within and 3 hospitals outside the study area). The exact admission time was routinely recorded for every acute stroke patient who presented to the ED. The time of onset of symptoms was obtained from the patient or an available witness and was considered as the time the patients or witnesses noticed the symptoms that may represent an initial stroke. If the symptoms occurred during sleep, the time of awakening was recorded as time of onset. Where patients were alone and history was unreliable, the time of symptom onset was considered to be the half-way point from when the patient was last seen well to the time the patient was found.^17^

### Statistical Analysis

The distribution of the delay from stroke onset to presentation at the ED was positively skewed and was summarized by the median and upper and lower quartiles. The differences in median time between sociodemographic and clinical subgroups were analyzed with the Mann-Whitney *U* test. Odds ratios (ORs) and 95% confidence intervals (CIs) were used to describe associations between prehospital delay (≥3 hours) and sociodemographic as well as clinical factors using multivariate logistic regression analyses. Covariables included in the logistic regression models were selected on the basis of their significance in univariable analysis (*P*<0.20) and their clinical relevance to the outcome of interest as reported from other studies. They included age, sex, ethnicity, socioeconomic status, NIHSS score, Glasgow Coma Score, stroke subtype, time of day, and year of stroke. All patients with onset of stroke between 2002 and 2010 were included in these analyses. Temporal changes in these associations were determined by examining the differences in the period immediately before and after the FAST campaign (ie, 2007/2008 versus 2009/2010). Logistic regression models were also used to examine the association between patient characteristics and the likelihood of receiving thrombolysis among a subset of patients with ischemic stroke occurring between 2002 and 2010. Patients with missing data were excluded from the logistic regression analysis. All analyses were performed with STATA 10.

### Ethical Approval

Patients or their relatives gave written informed consent to participate in the study. The design of the study was approved by the ethics committees of Guy's and St. Thomas' Hospital Trust, King's College Hospital, Queen's Square, and Westminster Hospital (London).

## Results

There were 2048 patients with a first-ever stroke registered between January 2002 and December 2010. Of these, 202 patients who had their strokes while in hospital and 454 with no data on time of stroke onset or arrival to hospital were excluded from the analyses. A total of 1392 patients were therefore included in the analyses. The mean age was 69.7 (standard deviation 15.2) years, and 712 (51.2%) of patients were men. There were no differences in age (*P*=0.82), sex (*P*=0.09), or ethnicity (*P*=0.44) between those with data on delay time and those without data, but a greater proportion of those without data had less severe strokes (45.9% compared with 25.1% in the lowest NIHSS quartile) at baseline (*P*<0.001).

Between 2002 and 2010 a total of 550 (39.5%) patients arrived within 3 hours of symptom onset, 712 (51.2%) within 4.5 hours, and 789 (56.7%) within 6 hours. There was no difference in the proportion of patients who arrived within 3 hours (*P*=0.30) in the period immediately before and after the FAST campaign (40.7% in 2007/2008 versus 44.9% in 2009/2010).

Table 1 shows the median prehospital delay time for all patients by selected sociodemographic and clinical characteristics. The overall median prehospital delay to presentation time was 4.73 (1.55–12.70) hours and was longer in patients of black ethnicity, patients living alone, patients with an ischemic stroke, and those with less severe strokes. There was, however, no significant difference in the median prehospital delay time for the period immediately before (2007/2008) and after (2009/2010) the FAST campaign.

**Table 1.** Median Time From Onset of Stroke to Arrival in ED by Selected Clinical and Sociodemographic Characteristics

		Median Time in Hours	
Characteristic	N (%)	(Q_1_–Q_3_)	*P*
All patients	1392	4.73 (1.55–12.70)	

Sex			

Male	712 (51.2)	4.90 (1.65–14.18)	0.29

Female	680 (48.8)	4.52 (1.50–11.60)	

Age, y			

<55	234 (16.8)	3.91 (1.27–12.84)	0.19

55–64	218 (15.7)	5.32 (1.50–16.41)	

65–74	339 (24.4)	5.20 (1.73–14.16)	

75–84	403 (29.0)	4.88 (1.64–12.56)	

≥85	198 (14.2)	4.15 (1.78–9.14)	

Ethnicity			

White	937 (68.6)	4.55 (1.50–12.49)	0.04

Black	332 (24.3)	5.20 (1.90–15.00)	

Other	97 (7.1)	3.52 (1.45–8.60)	

Socioeconomic status			

Nonmanual	251 (23.0)	5.16 (1.73–15.54)	0.16

Manual	550 (50.4)	5.20 (1.83–14.02)	

Economically inactive	290 (26.2)	4.01 (1.55–10.03)	

Prestroke living conditions			

Living alone	455 (32.7)	5.72 (1.97–13.69)	0.01

Living with others	802 (57.7)	4.01 (1.41–12.80)	

Other	133 (9.6)	4.97 (1.73–11.06)	

Type of stroke			

Infarct	1125 (80.8)	5.25 (1.78–14.20)	<0.001

PICH	195 (14.0)	2.51 (1.01–7.13)	

Unknown	72 (5.2)	3.98 (1.50–9.68)	

Glasgow Coma Scale			

<9	184 (13.4)	2.02 (0.98–6.28)	<0.001

9–15	1190 (86.6)	5.16 (1.78–15.00)	

Swallowing assessment			

Passed	814 (64.0)	6.35 (2.25–19.17)	<0.001

Failed	458 (36.0)	2.53 (1.08–6.75)	

NIHSS score in quartiles			

1 (least severe)	311 (25.1)	8.72 (3.14–22.55)	<0.001

2	325 (26.2)	6.00 (2.20–19.27)	

3	290 (23.4)	4.49 (1.50–11.53)	

4 (most severe)	314 (25.3)	2.32 (1.03–6.70)	

Time of day			

Daytime (6 am to 6 pm)	850 (61.1)	3.98 (1.50-11.55)	0.002

Nighttime (6 pm to 6 am)	542 (38.9)	6.47 (1.92–13.03)	

Day of the week			

Weekday	986 (70.8)	4.57 (1.50–12.49)	0.52

Weekend	406 (29.2)	4.99 (1.66–14.16)	

Year of stroke			

Pre-FAST (2007/2008)	329 (54.4)	4.22 (1.50–12.98)	0.38

Post-FAST (2009/2010)	276 (45.6)	3.87 (1.50–9.47)	

PICH indicates primary intracerebral hemorrhage.

In multivariable analysis shown in Table 2, the odds of presenting to hospital ≥3 hours after onset of stroke symptoms were increased in patients of black ethnicity compared with whites (OR: 1.63; 95% CI, 1.11–2.38). The odds of delay in presenting to hospital were significantly lower in those who lived with others (OR: 0.64; 95% CI, 0.45–0.89) and in those with more severe strokes characterized by the presence of dysphagia (OR: 0.66; 95% CI, 0.45–0.98) and higher NIHSS score (OR: 0.35; 95% CI, 0.20–0.61). Patients who had their stroke at night were more likely to arrive later than those whose strokes occurred during the day (OR: 1.85; 95% CI, 1.35–2.53). There was no difference in prehospital delay in the period immediately before the FAST awareness campaign compared with the postcampaign period (OR: 1.16; 95% CI, 0.65–2.08).

**Table 2.** Multivariable Analysis of Arrival to ED ≥3 Hours After Stroke

Characteristics	N (%)	OR (95% CI)	*P*
Sex			

Male	431 (60.6)	1.00	0.61

Female	410 (60.4)	1.08 (0.80–1.48)	

Age, y			

<55	132 (56.4)	1.00	0.25*

55–64	130 (59.9)	1.18 (0.65–2.15)	

65–74	213 (62.8)	1.14 (0.67–1.95)	

75–84	250 (62.0)	1.50 (0.88–2.57)	

≥85	116 (58.9)	1.24 (0.66–2.32)	

Ethnicity			

White	555 (59.4)	1.00	0.03

Black	217 (65.4)	1.63 (1.11–2.38)	

Other	54 (55.7)	0.95 (0.51–1.77)	

Socioeconomic status			

Nonmanual	158 (63.0)	1.00	0.90

Manual	356 (64.7)	0.97 (0.67–1.41)	

Economically inactive	164 (56.8)	1.32 (0.33–5.31)	

Prestroke living conditions			

Living alone	309 (68.1)	1.00	0.009

Living with others	453 (56.5)	0.64 (0.45–0.89)	

Other	78 (59.1)	1.13 (0.65–1.95)	

Type of stroke			

Ischemic	708 (63.1)	1.00	0.33

PICH	91 (46.7)	0.71 (0.44–1.13)	

Undefined	42 (58.3)	0.84 (0.41–1.70)	

Glasgow Coma Scale			

<9	78 (42.4)	1.00	0.54

≥9	752 (63.3)	0.83 (0.45–1.52)	

Swallowing assessment			

Passed	557 (68.5)	1.00	0.04

Failed	212 (46.4)	0.66 (0.45–0.98)	

NIHSS score in quartiles			

1 (least severe)	237 (76.2)	1.00	<0.001*

2	219 (67.6)	0.64 (0.41–1.00)	

3	175 (60.6)	0.54 (0.34–0.85)	

4 (most severe)	144 (45.9)	0.35 (0.20–0.61)	

Time of day			

Daytime (6 am to 6 pm)	485 (57.2)	1.00	<0.001

Night (6 pm to 6 am)	356 (65.7)	1.85 (1.35–2.53)	

Day of the week			

Weekday	598 (60.7)	1.00	0.72

Weekend	243 (60.0)	1.06 (0.76–1.48)	

Year of stroke			

Pre-FAST (2007/2008)	195 (59.5)	1.00	0.61

Post-FAST (2009/2010)	151 (54.9)	1.16 (0.65–2.08)	

OR indicates odds ratio; PICH, primary intracerebral hemorrhage.

* *P*-trend.

Among patients with ischemic stroke, 119 (11.0%) received thrombolysis. In multivariable analysis shown in Table 3, the odds of receiving thrombolysis increased with stroke severity characterized by higher NIHSS scores and decreased with increasing age (*P*-trend <0.001). The odds of receiving thrombolysis were also lower in patients of black ethnicity (OR: 0.49; 95% CI, 0.26–0.95). There was no difference in thrombolysis rates before or after the FAST campaign.

**Table 3.** Multivariable Analysis of Thrombolysis in Patients With Ischemic Stroke

Characteristics	N	Number of Patients Receiving Thrombolysis (%)	OR (95% CI)	*P*
Sex				

Male	564	53 (9.4)	1.00	0.61

Female	518	66 (12.7)	1.14 (0.68–1.92)	

Age, y				

<55	175	26 (14.9)	1.00	<0.001*

55–64	171	19 (11.1)	0.60 (0.26–1.37)	

65–74	263	28 (10.7)		

75–84	311	33 (10.6)	0.42 (0.20–0.89)	

≥85	162	13 (8.0)	0.29 (0.14–0.62)	

			0.17 (0.07–0.45)	

Ethnicity				

White	731	85 (11.6)	1.00	0.06

Black	252	23 (9.1)	0.49 (0.26–0.95)	

Other	77	10 (13.0)	1.23 (0.50–3.06)	

Socioeconomic status				

Nonmanual	179	14 (7.8)	1.00	0.96

Manual	351	20 (5.7)	0.94 (0.39–2.27)	

Economically inactive	286	50 (17.5)	0.79 (0.14–4.49)	

Prestroke living conditions				

Living alone	351	32 (9.1)	1.00	0.32

Living with others Other	608	74 (12.2)	1.03 (0.59–1.80)	

	122	13 (10.7)	0.54 (0.22–1.35)	

Glasgow Coma Scale				

< 9	85	13 (15.3)	1.00	0.12

≥ 9	966	105 (10.9)	1.98 (0.82–4.83)	

Swallowing assessment				

Passed	663	61 (9.2)	1.00	0.61

Failed	262	51 (19.5)	1.18 (0.63–2.21)	

NIHSS score in quartiles				

1 (least severe)	312	4 (1.3)	1.00	<0.001*

2	296	21 (7.1)	7.26 (2.05–25.73)	

3	210	39 (18.6)	23.23 (6.60–81.83)	

4 (most severe)	192	47 (24.5)	53.72 (13.68–210.97)	

Time of day				

Daytime (6 am to 6 pm)	623	88 (14.1)	1.00	<0.001

Night (6 pm to 6 am)	380	28 (7.4)	0.37 (0.21–0.64)	

Day of the week				

Weekday	777	92 (11.8)	1.00	0.16

Weekend	305	27 (8.9)	0.67 (0.38–1.18)	

Year of stroke				

Pre-FAST (2007/2008)	326	55 (16.9)	1.00	0.30

Post-FAST (2009/2010)	274	45 (16.4)	1.33 (0.77–2.30)	

OR indicates odds ratio.

A total of 251 (19.0%) patients died in hospital during their admission. In multivariable analyses adjusting for sociodemographic differences and case severity, the odds of death among those reporting to hospital later (≥3 hours) were not significantly different from those who arrived within 3 hours of symptom onset (OR: 0.90; 95% CI, 0.55–1.46).

## Discussion

This study found significant prehospital delays after onset of stroke symptoms similar to those reported from earlier national and international studies.^10,11,13,18,19^ Consistent with some prior studies, patients with more severe strokes, those with hemorrhagic stroke, those who lived with others, and those with daytime onset of symptoms were more likely to arrive within 3 hours of symptom onset.^11,18–21^ An important observation in this study was the increased prehospital delay observed in patients of black ethnicity in South London, similar to previous reports of black populations in the United States.^9–11^ There were suggestions that patients of black ethnicity were also less likely to receive thrombolysis in our study, possibly because they were more likely to arrive outside the therapeutic window currently approved for thrombolysis. Despite the higher incidence of stroke in patients of black ethnicity, previous studies have demonstrated that blacks are far less likely be aware of the availability of recombinant tissue plasminogen activator, independent of their level of education, and are also less likely to be aware of stroke warning symptoms and to call the emergency services.^15,22,23^ In a systematic review of mass media interventions designed to improve public recognition of stroke symptoms, emergency response, and early treatment, public education campaigns increased the awareness of stroke symptoms significantly but had limited impact on behavior.^24^ The Department of Health of England's multimedia “Stroke: Act FAST” campaign in 2009, aimed at raising public awareness of the symptoms of stroke and the importance of prompt treatment. Public education through mass media campaigns has been shown to increase awareness of stroke symptoms and reduce the delay in presentation and referral of stroke patients in some but not all studies.^11,23,25,26^ The direct impact of the FAST campaign in this multiethnic population is uncertain, as the study, designed before the campaign, did not specifically enquire about that. On a national level, however, an increase of 55.5% in stroke calls to emergency services in the first 4 months after the campaign was attributed to the campaign in a Department of Health report, and 84% of the public were noted to remember the campaign.^27^

The FAST campaign was aired for only a short time, which could have had an effect on the sustainability of any effects achieved. The findings from our population-based study suggest the need for more sustained educational campaigns targeted at those at higher risk of delay, including those of black ethnicity, who may not have been featured in the previous campaign. It is important for stroke awareness messages to encourage patients who develop symptoms to call the emergency services at any time of day as a matter of urgency. It is also necessary to have a better understanding of the symptoms experienced by patients and how patients evaluate the importance of these in terms of what action is deemed appropriate.

### Study Limitations

First, the number of patients excluded from the analyses because of missing data on delay time could potentially affect the interpretation of our results, yet the characteristics of patients with data on delay time did not differ significantly from those with complete data. Second, we did not collect information on other factors that could have influenced the prehospital delay, including distance from the hospital, mode of transport to the ED, awareness of stroke signs and symptoms, or patients' awareness and response specifically to the FAST campaign. Despite these limitations, our study was derived from a well-established stroke register of a multiethnic population with data collected prospectively on stroke severity and other factors and with the ability to study prehospital delay in different ethnic groups. The cohort design of this study enabled us to examine changes in prehospital delay time after the FAST campaign.

## Conclusions

In conclusion, this study demonstrated persistent significant prehospital delays after an acute stroke in this population and identified several factors, including black ethnicity, milder strokes, and living alone, that were associated with prehospital delay. Despite significant public education efforts in this study area by a national campaign to increase stroke awareness, there have been minimal changes in the response of patients to stroke symptoms, underscoring the need to identify factors that influence behavior, including time taken to evaluate symptoms perceived as minor, and address these with more effective public health programs. In addition, surveillance systems established to monitor and evaluate the effects of such programs in this population are required.
